# Patellar Tendon Allograft Reconstruction Using Achilles Graft With Traversing Suprapatellar Cerclage Limbs

**DOI:** 10.1002/atn2.70368

**Published:** 2026-10-30

**Authors:** Ronak J. Mahatme, Shawn A. Moore, Nishanth Muthusamy, Brian M. Grawe, Joseph D. Lamplot, David L. Bernholt

**Affiliations:** ^1^ Department of Orthopaedic Surgery University of Cincinnati College of Medicine Cincinnati Ohio U.S.A.; ^2^ Department of Orthopaedic Surgery Endeavor Health Medical Group Kildeer Illinois U.S.A.

## Abstract

Patellar tendon rupture and chronic extensor mechanism deficiency present significant reconstructive challenges, particularly in revision settings or when native tissue quality is poor. We describe a reproducible surgical technique for patellar tendon reconstruction using an Achilles allograft with traversing suprapatellar cerclage limbs. This construct helps facilitate controlled restoration of patellar height, immediate continuity of the extensor mechanism, and load sharing across the reconstruction during early healing.

## Graphical abstract

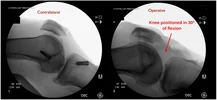

**VIDEO 1:** Step‐by‐step presentation of patellar tendon reconstruction using an Achilles tendon allograft with traversing suprapatellar cerclage augmentation performed with the patient in the supine position. The video illustrates the preoperative assessment of patellar height, preparation of the tibial tubercle trough, press‐fit fixation of the Achilles tendon allograft bone block, restoration of patellar height using controlled distal traction, proximal fixation through patellar bone tunnels and anchor augmentation, creation of crossed suprapatellar cerclage limbs, and final construct tensioning with the knee in full extension. The traversing cerclage construct serves as a load‐sharing internal brace intended to protect the reconstruction and reduce graft elongation during early rehabilitation. Video content can be viewed at https://doi.org/10.1002/atn2.70368.

Disruption of the knee extensor mechanism because of patellar tendon rupture results in profound functional impairment and disability.^1^ Although acute ruptures can be reliably treated with primary repair,^2^ chronic injuries, revision cases, and situations involving poor tissue quality remain challenging. Tendon retraction, scarring, patellar height abnormalities, and compromised tendon biology frequently preclude a successful repair in these settings.^3^

Allograft reconstruction provides a reliable salvage option for chronic and revision extensor mechanism injuries, particularly in the setting of insufficient native tendon, providing sufficient graft length and strength while avoiding donor‐site morbidity.^4^ However, the optimal reconstruction strategy remains unclear. A systematic review comparing outcomes across graft types used for chronic patellar tendon reconstruction showed substantial heterogeneity and low levels of evidence, precluding consensus on a superior technique.^5^ Further, published case series continue to report postoperative extensor lag of 5° to 10°,^4^, ^6^ underscoring the importance of secure fixation, appropriate tensioning, and protection of the reconstruction during early healing.

We describe a technique for patellar tendon allograft reconstruction augmented with traversing suprapatellar cerclage limbs. This construct functions as a load‐sharing internal brace, facilitates accurate restoration of patellar height, and protects the allograft during the vulnerable early postoperative period.

## SURGICAL TECHNIQUE

Preoperatively, magnetic resonance imaging is used to assess tendon quality, tear morphology, and the degree of retraction. In the operating room, the patient is positioned supine on the operating room table. A lateral view fluoroscopic image of the contralateral knee obtained at approximately 30° of flexion is recommended to establish a target patellar height for intraoperative restoration (Figure 1). To standardize knee flexion during imaging, a triangle knee positioner may be used (Figure 2). The positioner is placed under the contralateral knee before preparation of the operative extremity to obtain the reference image and is then applied to the operative knee intraoperatively to reproduce the same degree of flexion, allowing accurate comparison of patellar height.

**FIGURE 1:**
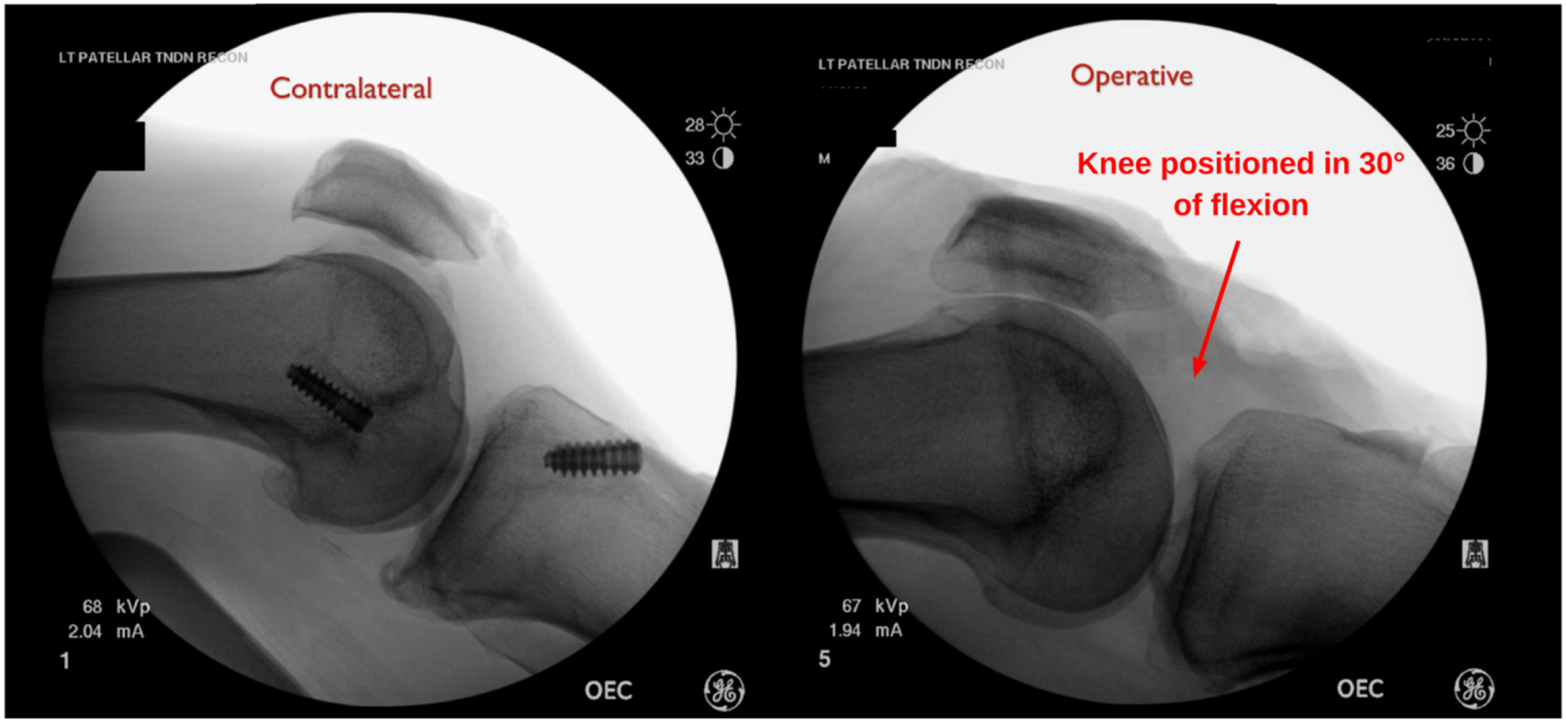
Lateral fluoroscopic image of the contralateral (reference) knee and ipsilateral (operative) knee obtained with the patient in the supine position and both knees positioned at approximately 30° of flexion. These images are obtained before reconstruction to establish the target patellar height for intraoperative restoration of extensor mechanism length and sagittal patellar alignment.

**FIGURE 2:**
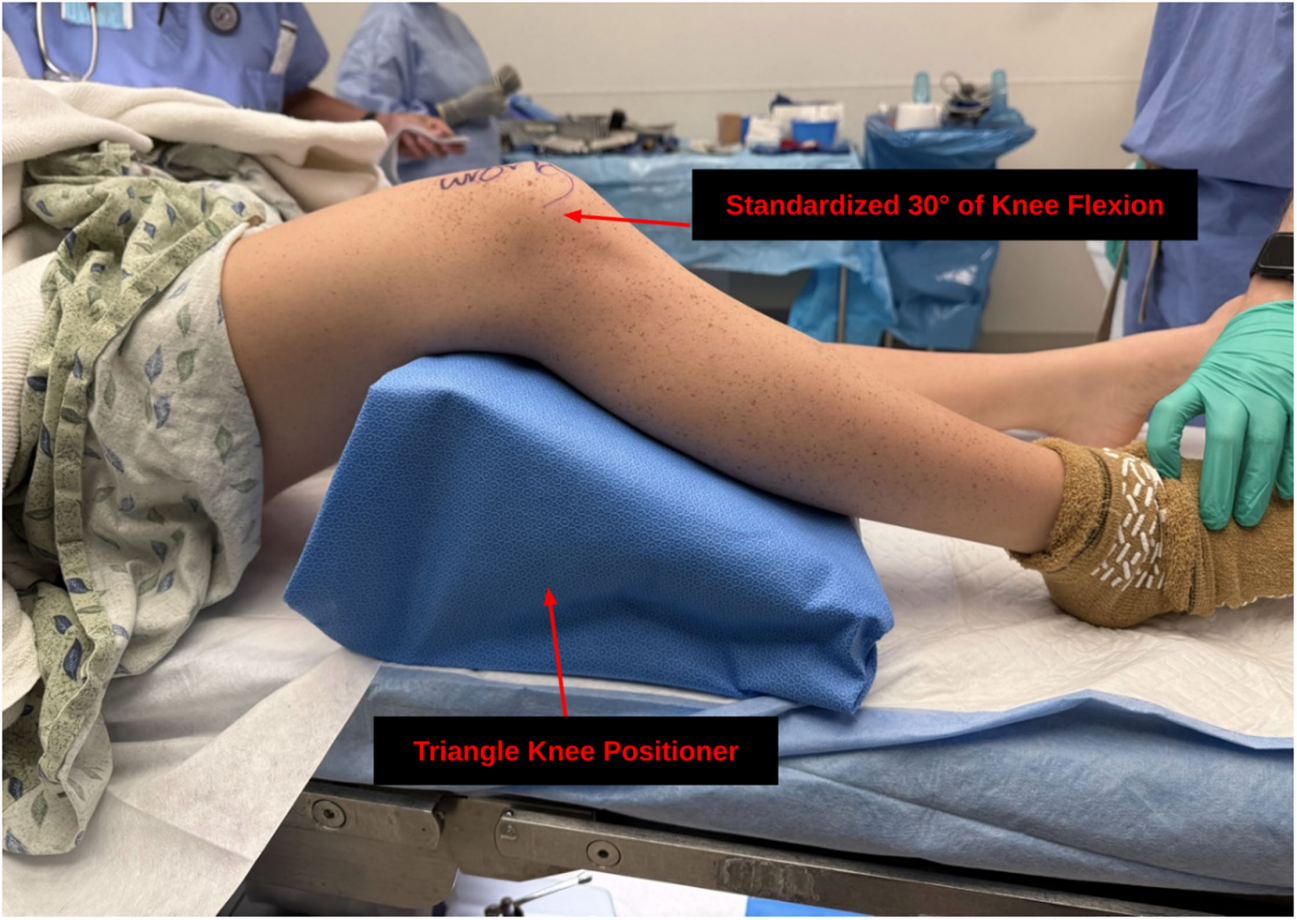
Intraoperative setup showing the use of a triangle knee positioner with the patient in the supine position to standardize knee flexion during fluoroscopic imaging. The same degree of flexion is reproduced between the contralateral and operative extremities to permit accurate intraoperative assessment and restoration of patellar height.

### Surgical Approach

We describe in detail the procedure as seen in Video 1. A midline incision is made from the tibial tubercle to the proximal pole of the patella. Full‐thickness flaps are elevated, and the fibrous tissue is excised to expose the tibial tubercle and distal pole of the patella. The scar tissue and nonviable tendon are debrided to assess the quality and mobility of the remaining extensor mechanism. The tibial tubercle is prepared, and a bone trough measuring approximately 25 mm in length and 10 to 12 mm in width and depth is created (Figure 3).

**FIGURE 3:**
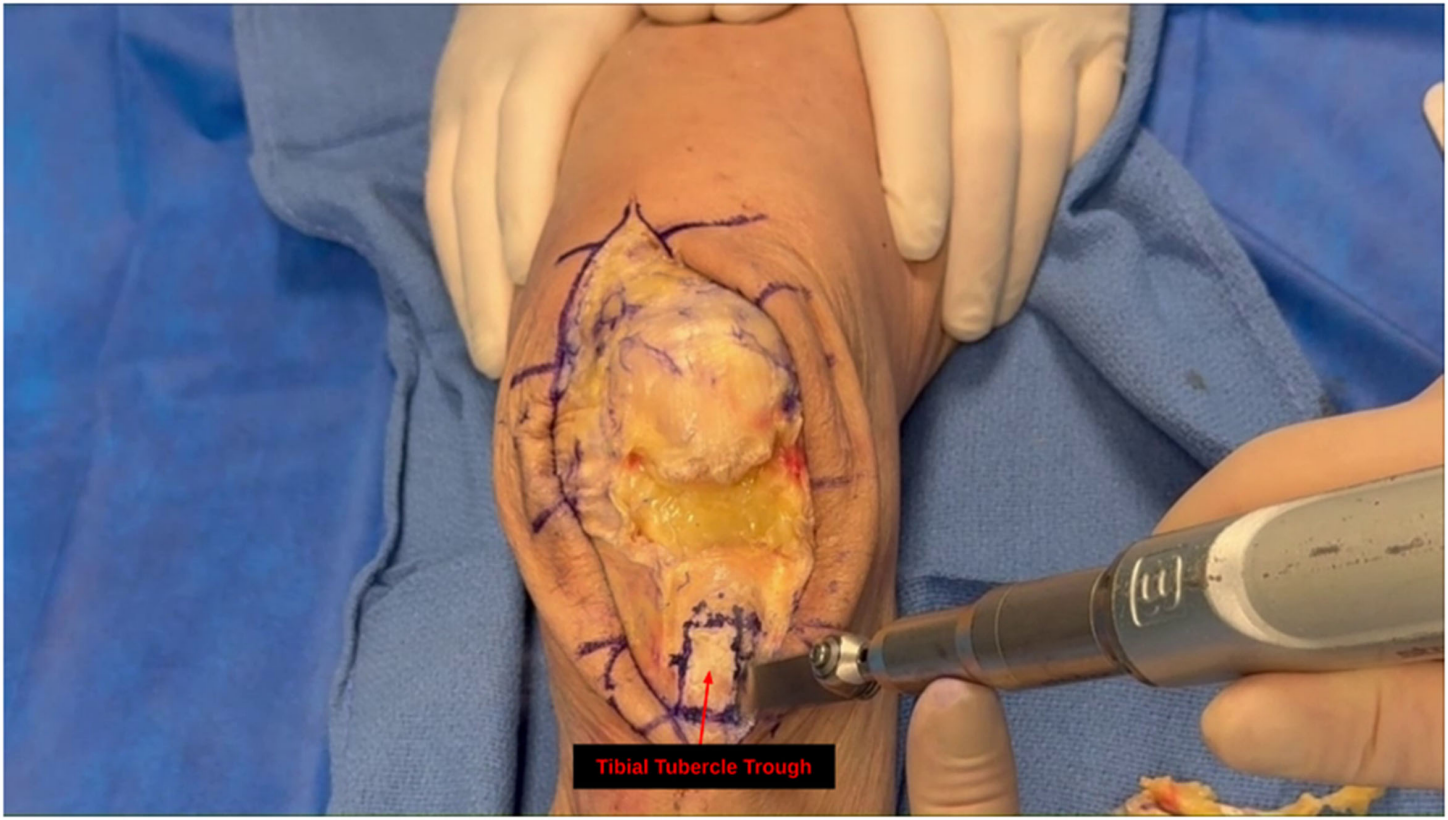
Intraoperative photograph of the operative knee with the patient in the supine position showing the preparation of the tibial tubercle. A rectangular trough measuring approximately 25 mm in length and 10 to 12 mm in width and depth is created to accept the Achilles tendon allograft bone block and provide stable distal fixation for reconstruction of the extensor mechanism.

### Graft Preparation

An Achilles tendon allograft is prepared on the back table. The bone plug is measured, shaped, and contoured to allow a press fit within the tibial tubercle trough. The graft is then tamped into the trough. After press‐fit placement of the bone plug, supplemental fixation is achieved using two 3.5‐mm or 4.0‐mm cannulated screws (Figure 4). Fixation is confirmed to be stable and screws of appropriate length prior to proceeding with the proximal portion of the reconstruction.

**FIGURE 4:**
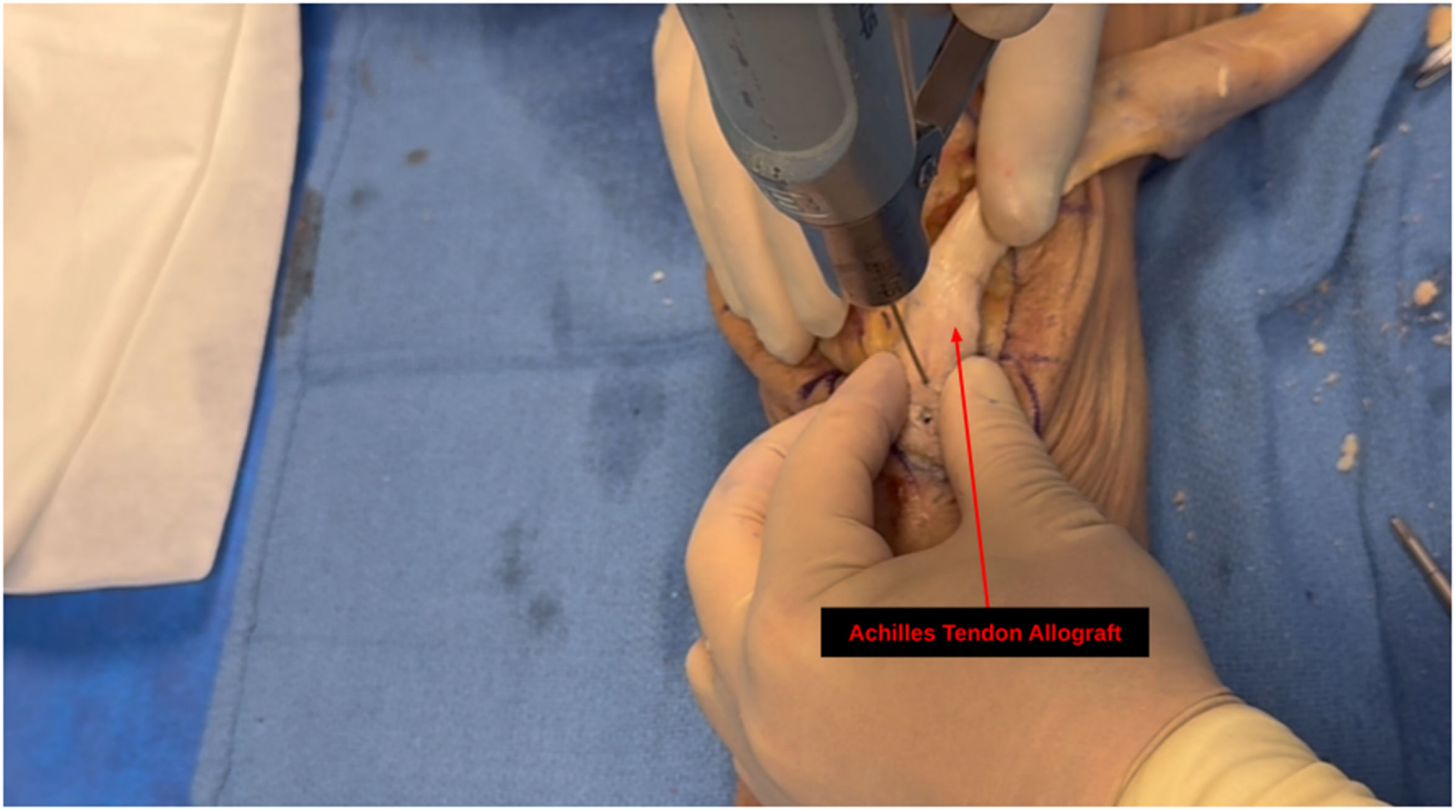
Intraoperative photograph of the operative knee with the patient in the supine position showing the press‐fit insertion of the Achilles tendon allograft bone plug into the prepared tibial tubercle trough. Supplemental fixation is achieved using two 4.0‐mm cannulated screws to establish secure distal fixation prior to proximal graft tensioning and find patellar height restoration.

Small rents are sharply created in the medial and lateral retinaculum adjacent to the quadriceps tendon. A hemostat is used to spread through soft tissue and create a suprapatellar tract, and a tape‐style suture is passed through this tract just proximal to the patella. Traction is applied distally to mobilize the patella and restore patellar height (Figure 5), guided by fluoroscopic comparison to preoperative imaging of the contralateral knee.

**FIGURE 5:**
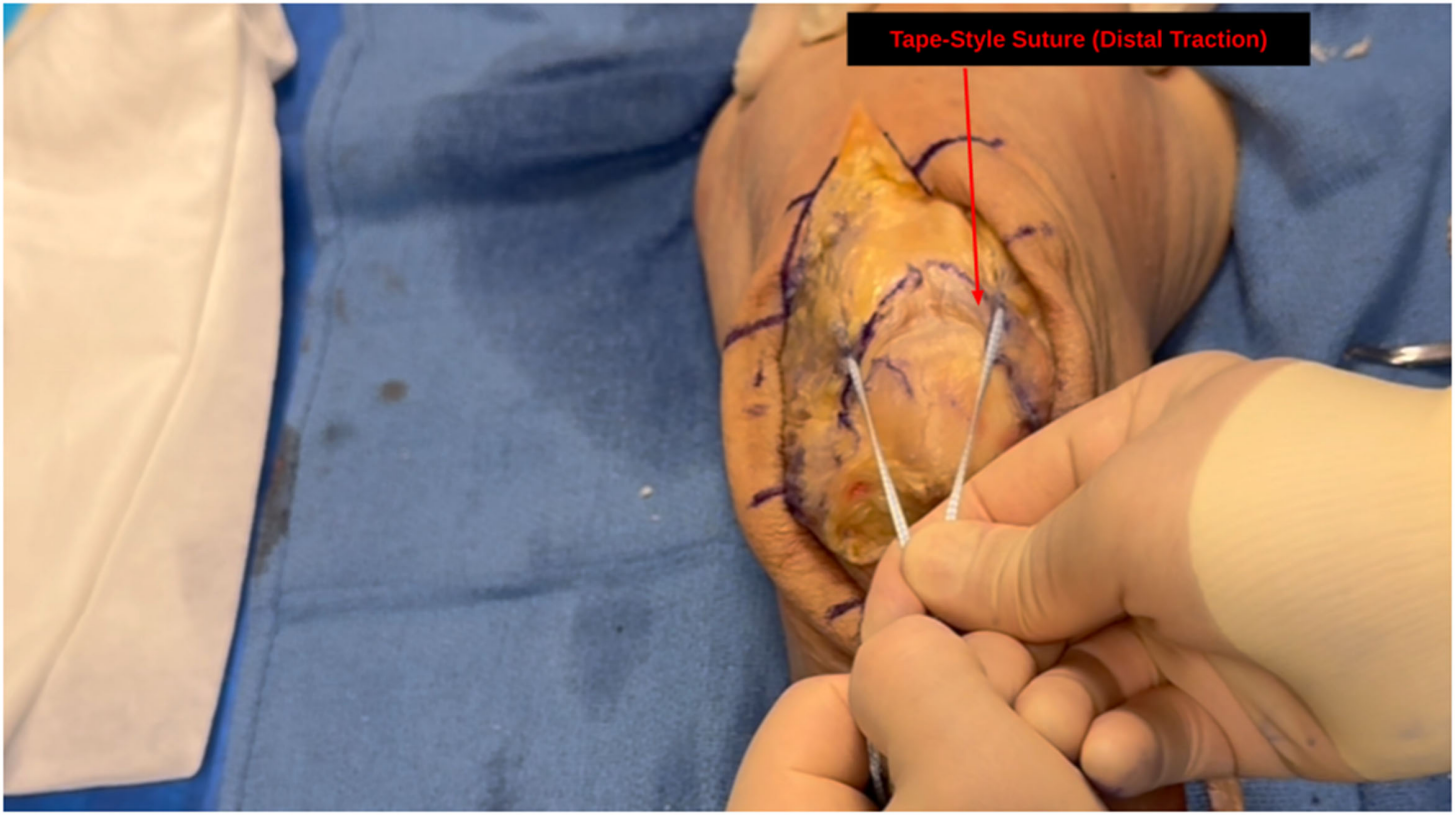
Intraoperative photograph of the operative knee with the patient in the supine position showing the distal traction applied through tape‐style suture passed across the suprapatellar interval. Controlled distalization of the patella is performed under fluoroscopic guidance using comparison to the contralateral knee to restore native patellar height and minimize postoperative extensor lag.

Once an appropriate patellar height is achieved, the allograft is marked at the point corresponding to contact with the distal pole of the patella. The distal patella is prepared, and Krackow sutures are placed in the graft, with sutures exiting the tendon at the marked level. Two separate #5 FiberWire sutures made by Arthrex are used to create medial and lateral suture sets, with the limbs exiting on the deep surface of the graft (Figure 6).

**FIGURE 6:**
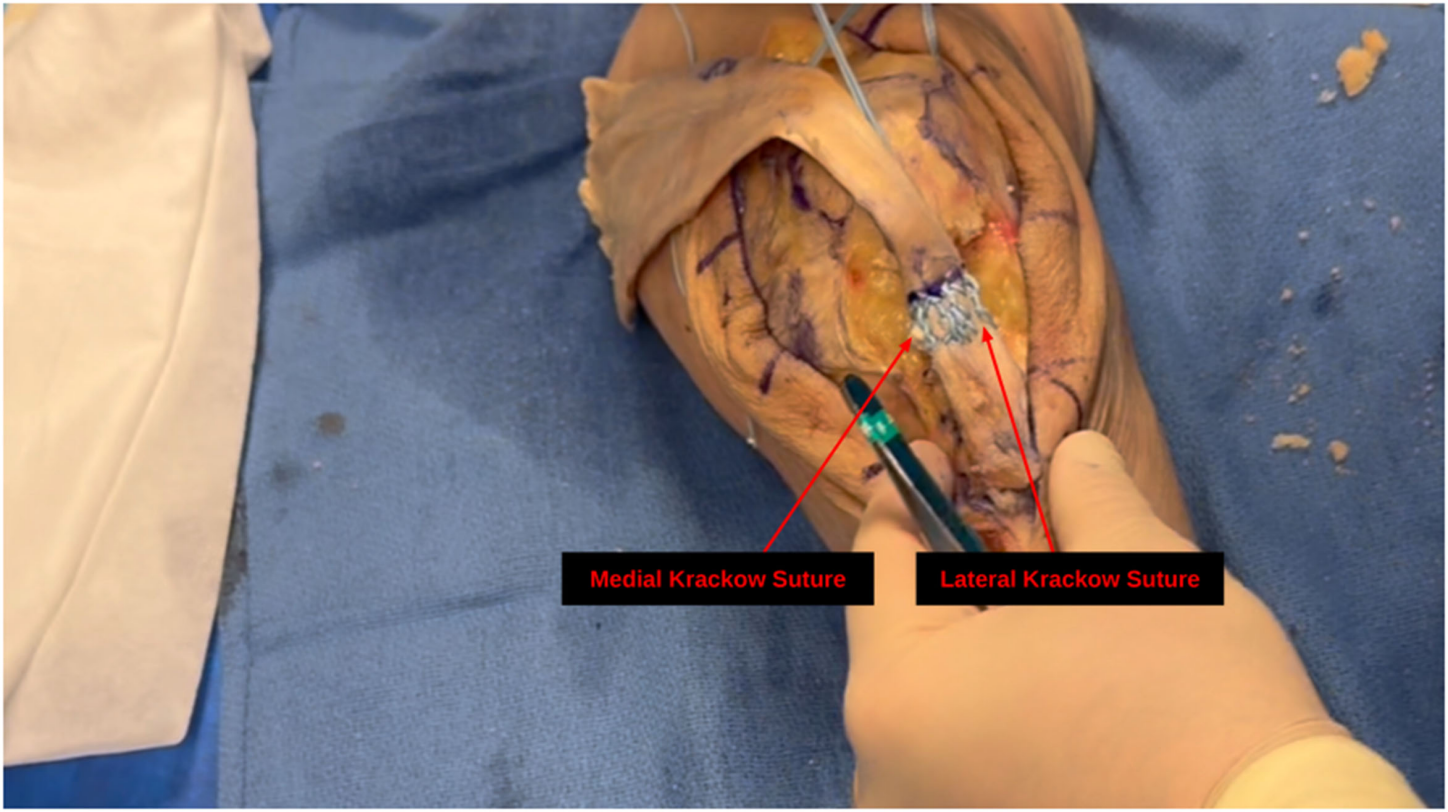
Intraoperative photograph of the knee with the patient in the supine position showing the placement of medial and lateral Krackow suture constructs within the Achilles tendon allograft. Suture limbs exit at the level corresponding to contact with the distal pole of the patella to allow controlled tensioning and anatomic fixation of the reconstruction.

### Patellar Fixation

Bone tunnels are created through the patella in standard fashion using Beath pins. A central pin is placed, followed by a medial pin to define available bone stock for secondary fixation using an Arthrex 1.9‐mm all‐suture FiberTak Biceps Anchor. The chuck of the Beath pin is used to stabilize the patella during anchor insertion, facilitating secure placement. Once the medial anchor is seated, the medial Krackow suture limb is passed through the medial bone tunnel (Figure 7).

**FIGURE 7:**
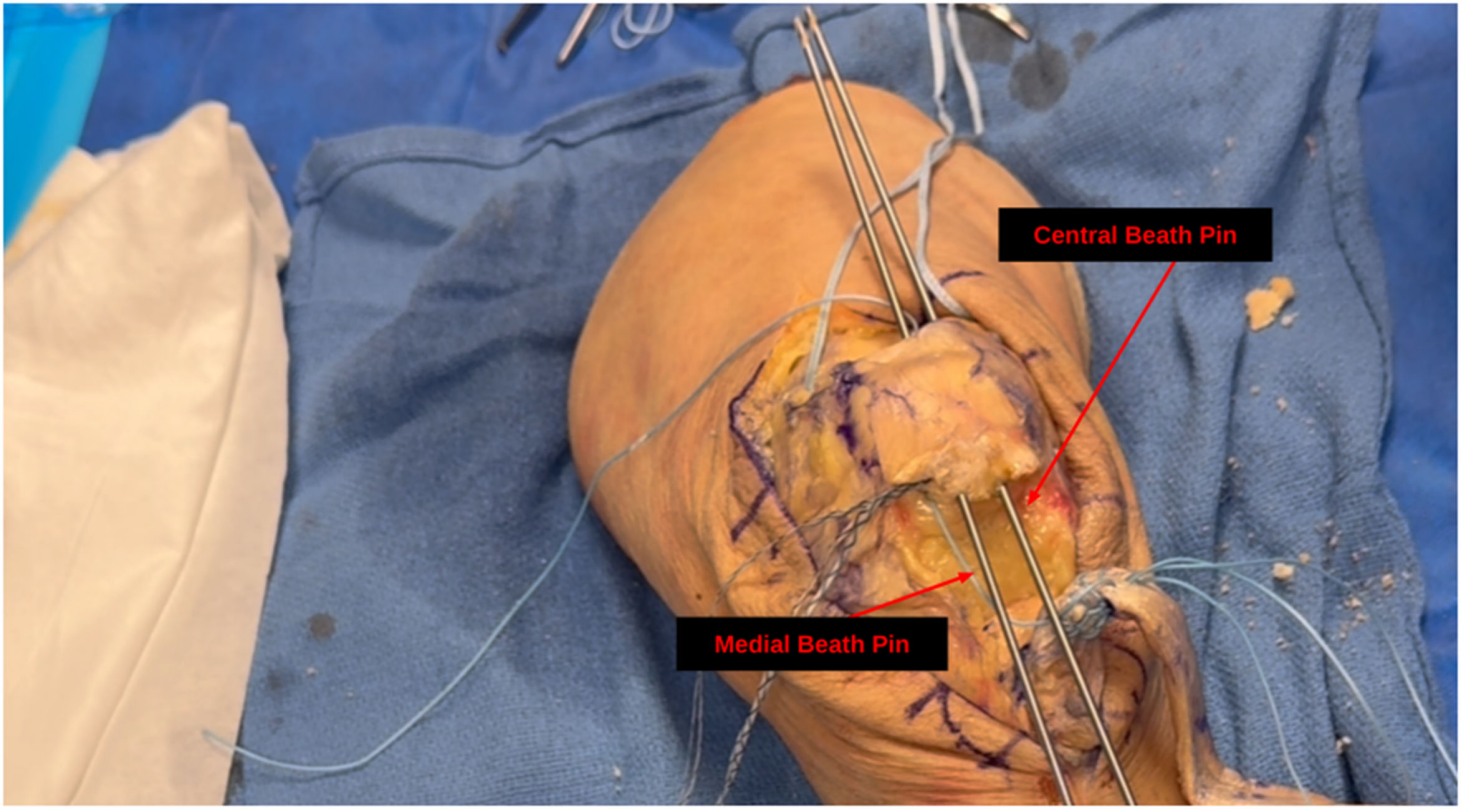
Intraoperative photograph of the operative knee with the patient in the supine position showing the creation of patellar bone tunnels using Beath pins. A central pin is placed first, followed by the placement of a medial pin to define available patellar bone stock for secondary fixation with a 1.9‐mm all‐suture anchor.

A lateral bone tunnel is then drilled, a second FiberTak Biceps Anchor is placed (between central and lateral bone tunnels), and the lateral Krackow suture limb is then passed. The central sutures from the graft are passed using a wire suture passer because the bone tunnel diameter makes it difficult to pass the sutures directly using the Beath pin. Before tying the Krackow suture sets over the proximal patella, anchor sutures are sewn into the patellar tendon just proximal to the Krackow exit points, passed from deep to superficial, and tied securely with the knee in full extension. Bone tunnel sutures are then tied over the proximal pole of the patella. All sutures are cut after fixation is confirmed.

### Creation of Traversing Cerclage Limbs

The graft is trifurcated to create a central limb and 2 peripheral limbs, which will be used to create traversing suprapatellar cerclage limbs (Figure 8). The medial and lateral limbs are prepared to a size of approximately 5 mm in diameter. An Arthrex looped FiberTape suture is used to shuttle 1 graft limb across the suprapatellar tract. The lateral limb is passed medially, and a second passing suture is used to pull the medial limb laterally, creating crossed suprapatellar cerclage limbs (Figure 9).

**FIGURE 8:**
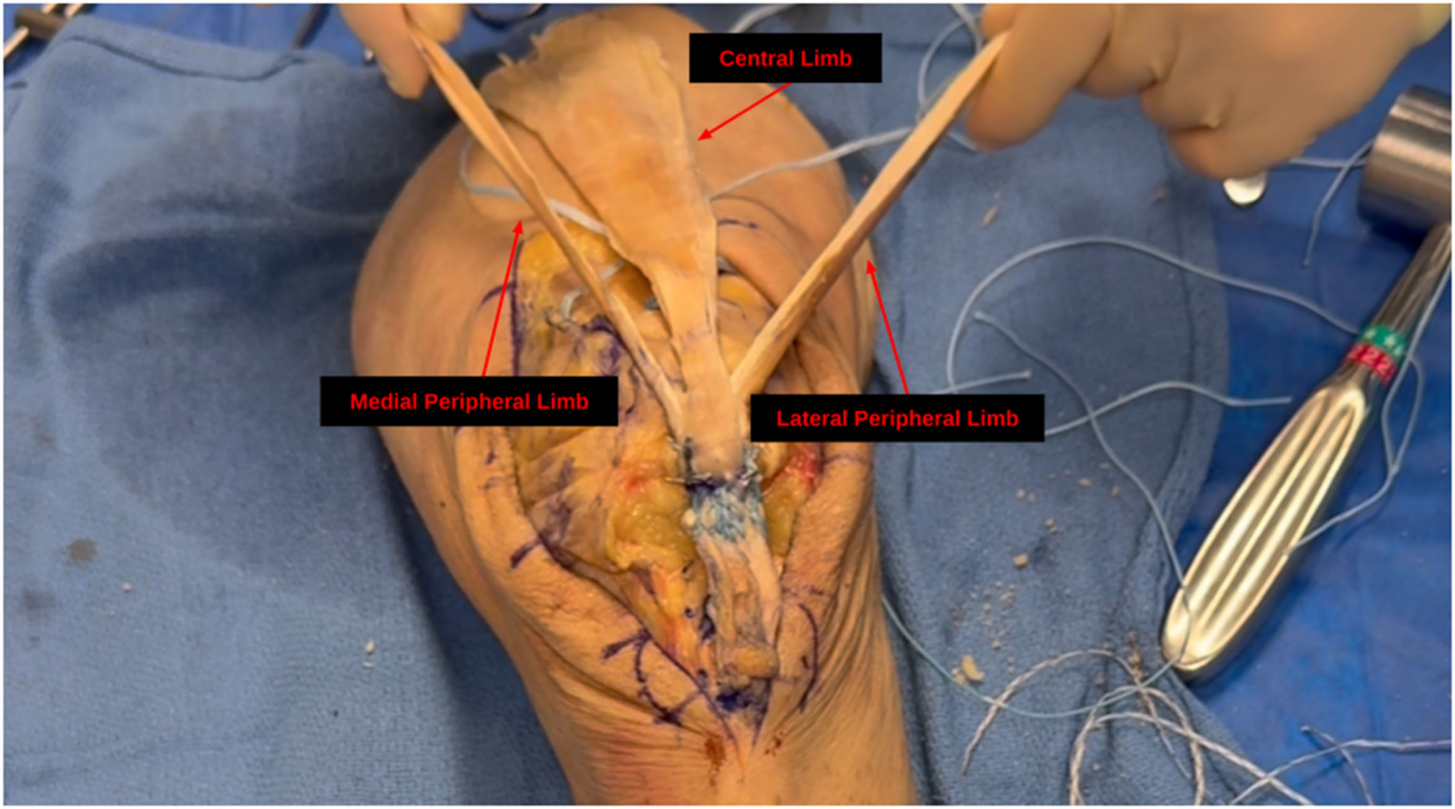
Intraoperative photograph of the operative knee with the patient in the supine position showing the trifurcation of the Achilles tendon allograft into 1 central limb and 2 peripheral limbs. The peripheral limbs are prepared to create traversing suprapatellar cerclage limbs that augment the reconstruction and function as a load‐sharing internal brace during early healing.

**FIGURE 9:**
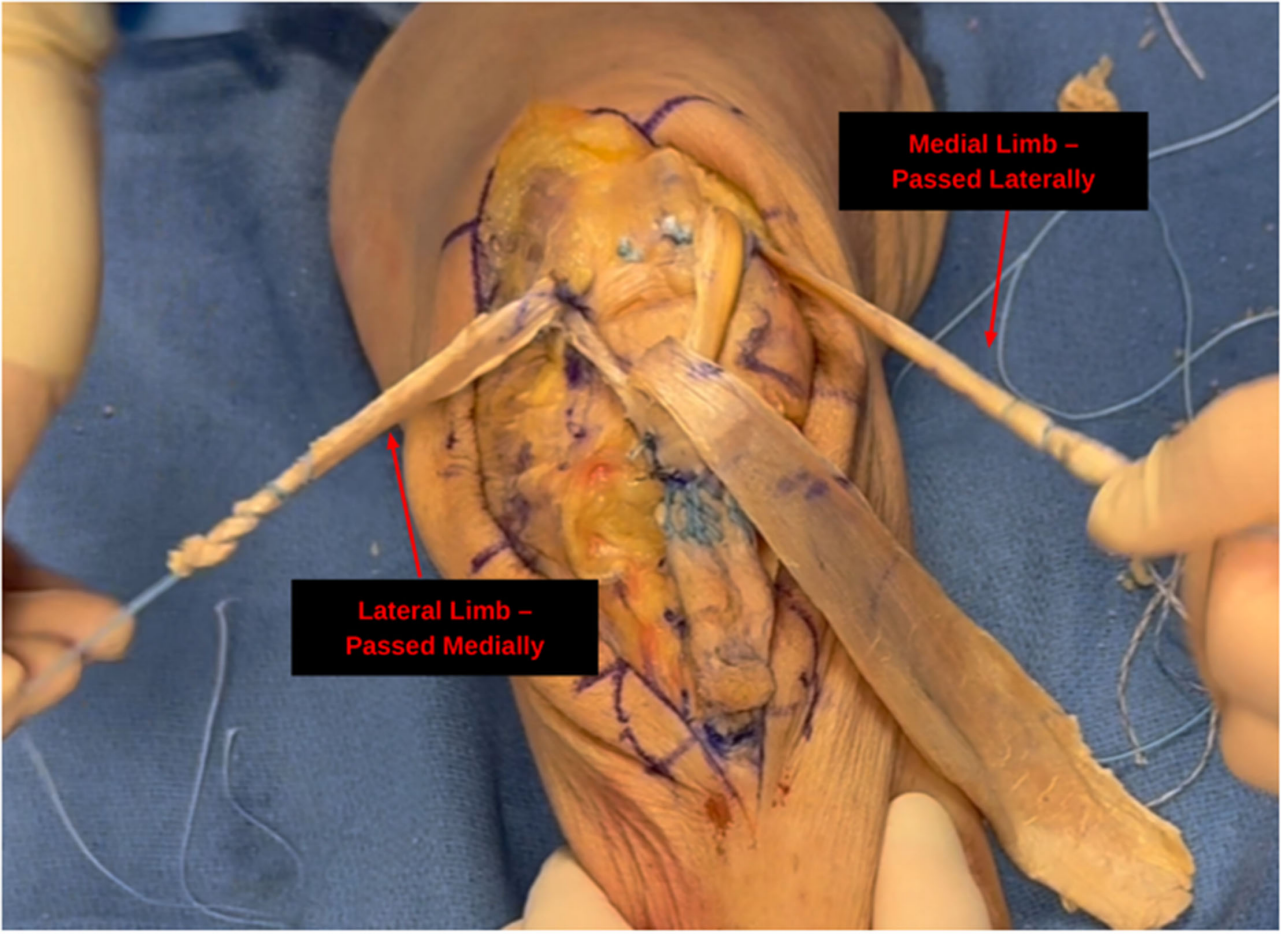
Intraoperative photograph of the operative knee with the patient in the supine position showing the creation of crossed suprapatellar cerclage limbs by passing the lateral limb medially and medial graft limb laterally through the suprapatellar interval. This crossed configuration reinforces the reconstruction, assists maintenance of patellar height, and protects against graft elongation during postoperative rehabilitation.

Distal traction is applied, and each graft limb is secured at its retinacular exit point with sutures placed directly through the graft and surrounding soft tissue. The central limb is secured to the quadriceps tendon and overlying patellar periosteum, and excess graft is excised. The 2 traversing limbs are then brought distally and fixed near the level of the tibial tubercle using 2 separate suture anchors, 1 on each side of the tubercle (Figure 10). Final tensioning is performed with the knee in full extension.

**FIGURE 10:**
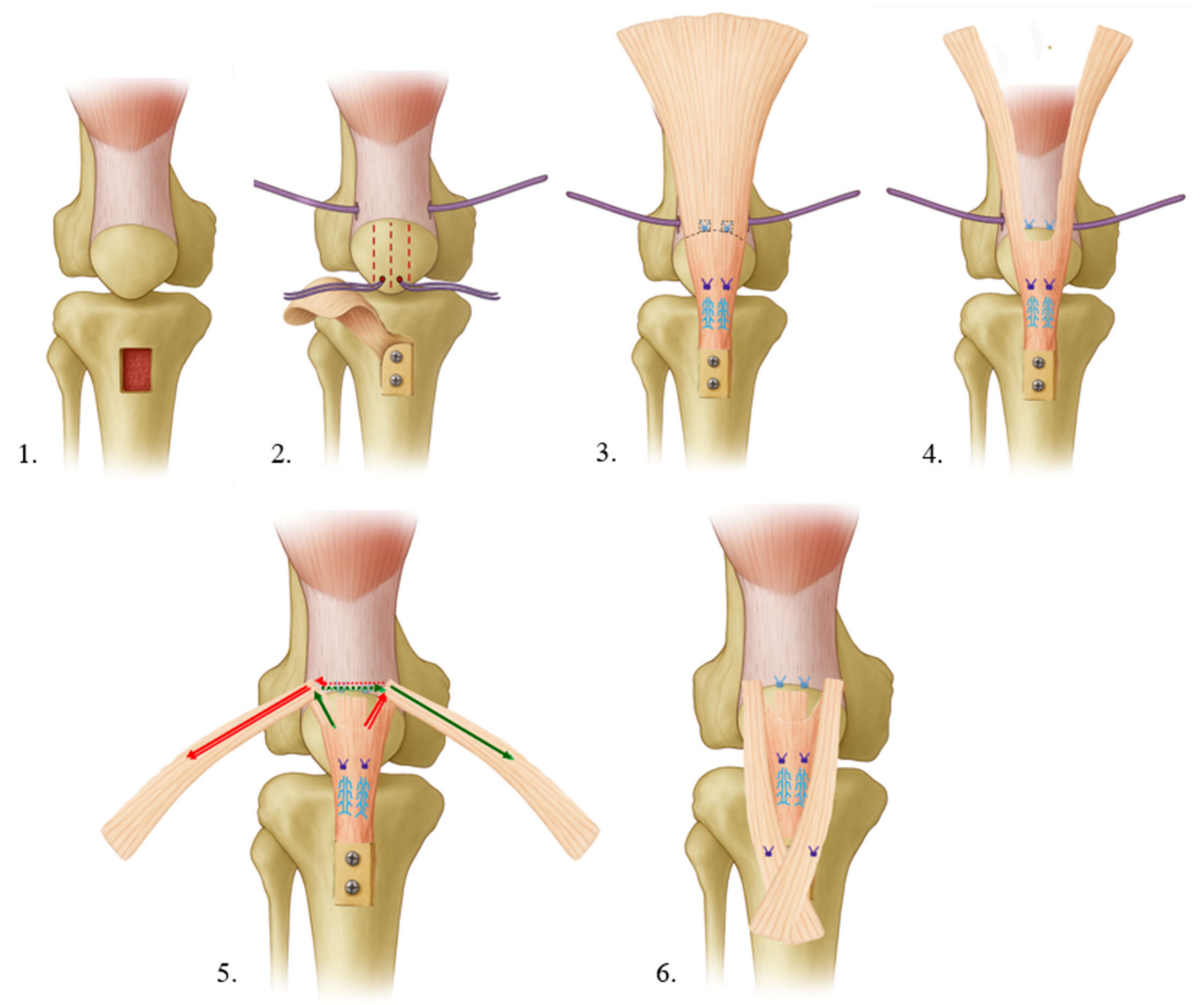
Step‐by‐step schematic of patellar tendon allograft reconstruction using Achilles tendon allograft with traversing suprapatellar cerclage augmentation: (1) preparation of the tibial tubercle trough; (2) press‐fit insertion and screw fixation of the Achilles bone plug with restoration of patellar height; (3) proximal graft fixation through patellar bone tunnels with anchor augmentation; (4) graft trifurcation; (5) creation of crossed suprapatellar cerclage limbs; and (6) distal fixation of cerclage limbs adjacent to the tibial tubercle. The cerclage construct functions as a protective load‐sharing augmentation to support the reconstruction during early rehabilitation.

### Postoperative Rehabilitation Protocol

Patients are placed in a hinged knee brace locked in extension with protected weight‐bearing for the first 4 weeks. From weeks 4 to 6, weight‐bearing as tolerated is permitted with the brace locked in extension. The controlled range of motion from 0° to 30° is allowed during the first 2 to 3 weeks, followed by progressive increases of approximately 20° per week, with a goal of achieving 90° of flexion by 6 weeks postoperatively. Representative preoperative and postoperative radiographs showing the restoration of patellar height are shown in Figure 11.

**FIGURE 11:**
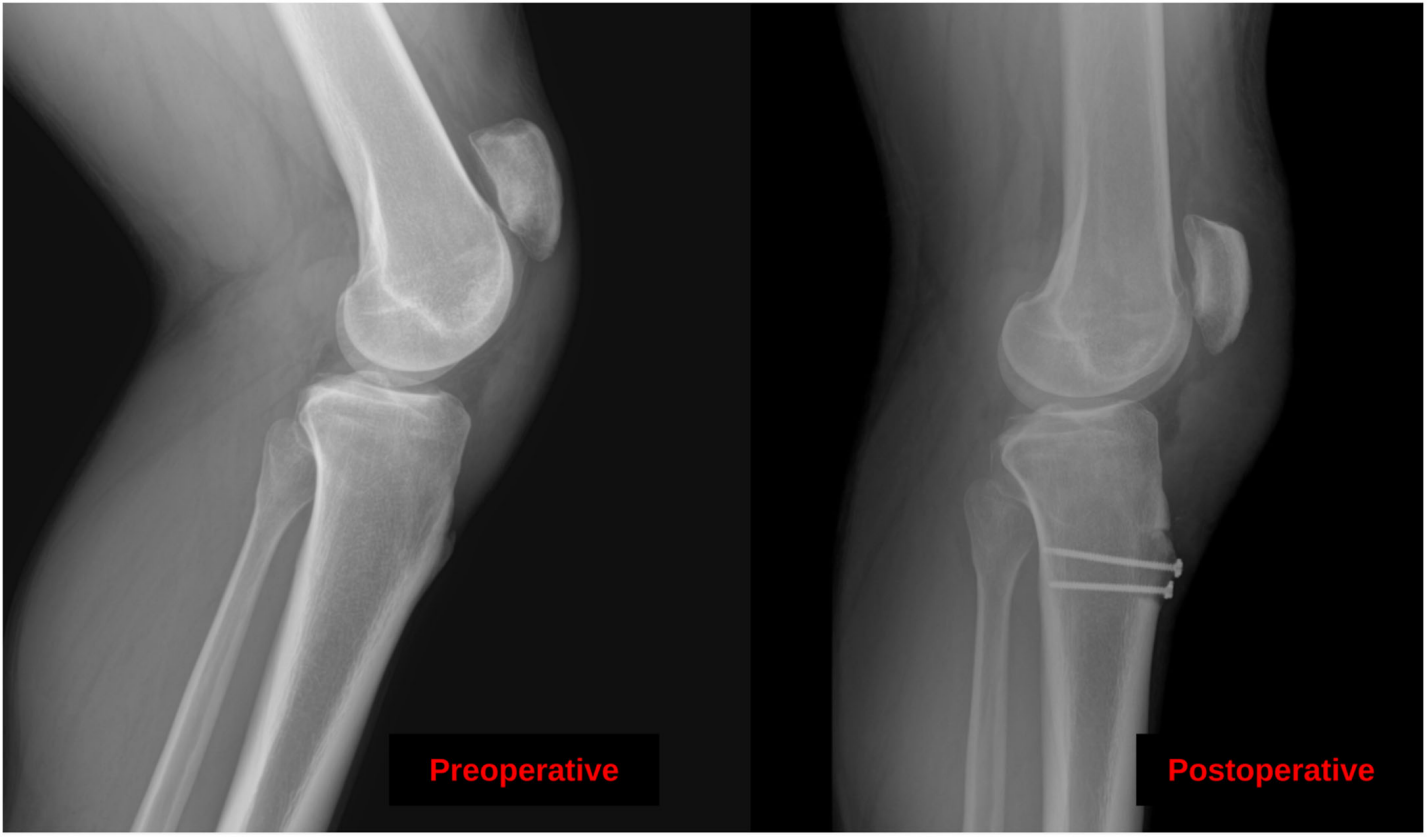
Representative lateral radiographs of the operative knee obtained before and after reconstruction. The preoperative image shows chronic patellar tendon rupture with patella alta and disruption of extensor mechanism continuity, whereas the postoperative image shows the restoration of patellar height and successful reconstruction using Achilles tendon allograft with traversing suprapatellar cerclage augmentation.

## DISCUSSION

Patellar tendon allograft reconstruction has emerged as an important salvage procedure for chronic and revision extensor mechanism injuries.^4^ However, prior techniques vary widely with respect to graft choice, fixation strategy, and postoperative rehabilitation, and no single method has shown superior outcomes.^3^, ^5^

In this technical note, we describe a patellar tendon allograft reconstruction augmented with traversing suprapatellar cerclage limbs. The distinguishing feature of this technique is the use of crossed suprapatellar graft limbs functioning as a load‐sharing internal brace. This construct assists in controlled distalization of the patella, protects the primary reconstruction during early motion, and may mitigate graft elongation, which is a known contributor to postoperative extensor lag (Table 1).

**TABLE 1:** Advantages and Disadvantages of Patellar Tendon Allograft Reconstruction With Suprapatellar Traversing Cerclage

**Advantages**	**Disadvantages**
Reconstructive option when primary repair is not feasible	Increased cost
Applicable to chronic and revision extensor mechanism deficiencies	Potential for allograft‐related complications (graft resorption, inflammatory reaction, and infection)
Addresses compromised native tendon biology	Less suitable for acute ruptures with good quality tissue
Allows simultaneous reconstruction and reinforcement of the extensor mechanism	Limited long‐term outcome data
Can be used in complex post‐total knee arthroplasty cases	

Several technical considerations are critical to the successful execution of this technique (Table 2). The restoration of patellar height is guided by contralateral knee imaging and confirmed intraoperatively prior to definitive fixation. Tensioning is performed with the knee in full extension because inadequate tension has been associated with increased postoperative extensor lag in prior series.^3^, ^4^ In chronic cases, aggressive mobilization of the extensor mechanism is often required to allow anatomic reduction without excessive graft tension. The suprapatellar cerclage limbs provide additional protection during early rehabilitation, permitting a controlled range of motion while minimizing stress on the primary graft fixation.

**TABLE 2:** Pearls and Pitfalls of Patellar Tendon Allograft Reconstruction With Suprapatellar Traversing Cerclage

**Pearls**	**Pitfalls**
Obtain contralateral knee radiographs to determine target patellar height	Failure to restore anatomic patellar height, leading to extensor lag
Mobilize extensor mechanism in chronic cases	Inadequate mobilization resulting in excessive graft tension
Tension the reconstruction with the knee in full extension	Tensioning with the knee flexed, resulting in patella alta
Confirm patellar tracking and height before final fixation	Failure to reassess patellar position after fixation
Use suprapatellar cerclage limbs to protect the graft during early healing	Relying on cerclage limbs as the primary fixation construct
Advance rehabilitation in a controlled, staged manner	Premature aggressive rehabilitation leading to graft elongation

As with any allograft reconstruction, this technique carries inherent risks, including increased cost,^7^ risk of infection, graft resorption, and inflammatory reaction^8^ (Table 1). These risks may be amplified in post‐total knee arthroplasty patients and should be considered when selecting candidates for reconstruction. Additionally, failure to adequately tension the construct or premature advancement of rehabilitation may compromise outcomes (Table 2).

In conclusion, patellar tendon allograft reconstruction with suprapatellar traversing cerclage limbs is a versatile and reproducible salvage technique for chronic and revision extensor mechanism injuries. By emphasizing anatomic restoration of the patellar height, secure fixation, and graft protection during early healing, this approach may reduce extensor lag and improve functional outcomes in a challenging patient population.

## DISCLOSURES

The authors (R.J.M., S.A.M., N.M., B.M.G., J.D.L., D.L.B.) declare that they have no known competing financial interests or personal relationships that could have appeared to influence the work reported in this article.
